# A method of localization and segmentation of intervertebral discs in spine MRI based on Gabor filter bank

**DOI:** 10.1186/s12938-016-0146-5

**Published:** 2016-03-22

**Authors:** Xinjian Zhu, Xuan He, Pin Wang, Qinghua He, Dandan Gao, Jiwei Cheng, Baoming Wu

**Affiliations:** State Key Laboratory for Trauma, Burn and Combined Injury, Fifth Department, Research Institute of Field Surgery, Daping Hospital, Third Military Medical University of Chinese PLA, Chongqing, 400042 China; College of Communication Engineering, Chongqing University, Chongqing, 400044 China; Department of Orthopaedics, 113th Hospital, Ningbo, 315040 Zhejiang China

**Keywords:** Magnetic resonance image, Intervertebral disc, Localization, Segmentation, Gabor filter

## Abstract

**Background:**

Spine magnetic resonance image (MRI) plays a very important role in the diagnosis of various spinal diseases, such as disc degeneration, scoliosis, and osteoporosis. Accurate localization and segmentation of the intervertebral disc (IVD) in spine MRI can help accelerate the diagnosis time and assist in the treatment by providing quantitative parameters. In this paper, a method based on Gabor filter bank is proposed for IVD localization and segmentation.

**Methods:**

First, the structural features of IVDs are extracted using a Gabor filter bank. Second, the Gabor features of spine are calculated and spinal curves are detected. Third, the Gabor feature images (GFI) of IVDs are calculated and adjusted according to the spinal curves. Fourth, the IVDs are localized by clustering analysis with GFI. Finally, an optimum grayscale-based algorithm with self-adaptive threshold, combined with the localization results and Gabor features of the spine, is performed for IVDs segmentation.

**Results:**

The proposed method is verified by an MRI dataset consisting of 278 IVDs from 37 patients. The accuracy of localization is 98.23 % and the dice similarity index for segmentation evaluation is 0.9237.

**Conclusions:**

The proposed Gabor filter based method is effective for IVD localization and segmentation. It would be useful in computer-aided diagnosis of IVD diseases and computer-assisted spine surgery.

## Background

Spinal diseases, such as low-back pain (LBP), are common chronic diseases and are harmful to human health. LBP is a common symptom with considerable social and economic repercussions [1]. LBP is experienced by 25 to 50 % of the adult population in the United States. The healthcare costs for spine pain (mainly for LBP) is increasing every year in the United States [2]. The degeneration of intervertebral discs (IVDs) is the main factor resulting in chronic LBP and disability [3]. Analysis of magnetic resonance imaging (MRI), including disc localization and segmentation, is the main tool to assess degenerative disk disease and evaluate the spinal cord, ligaments, and lesions, as it provides high-resolution, high-contrast images in serial contiguous slices [4].

Previously, disc localization and segmentation in spine MRI was performed by the radiologists manually, and the results depended on the a priori knowledge and experience. Additionally, manual localization and segmentation is laborious and lacks reproducibility between observers. Therefore, it is necessary to develop methods to localize and segment the IVDs automatically. An accurate localization and segmentation with computer-aided diagnosis (CAD) for discs in spine MRI would be useful in the quantification of disc degeneration, diagnosis of the disease, and computer-assisted spine surgery [5–7]. But, the differences in size, shape, appearance, intensity of different IVDs, and the ambiguous IVD boundaries and similar intensity with surrounding tissues may increase the difficulty of recognition.

Model-based methods are often used to analyze IVDs in past years. Alomari et al. [6] proposed a two-level probabilistic model for localization of discs from MRI. Michopoulou et al. [7] also presented a semiautomatic approach to segment both normal and degenerated lumbar IVDs. Peng et al. [8] used a model-based searching method to localize whole spine discs. Castro et al. [9] segmented the IVDs using active contour models and fuzzy C-means. Haq et al. [10] proposed a segmentation approach based on the discrete simplex surface model. Law et al. [11] employed a novel anisotropic-oriented flux model to segment the IVDs. These methods are effective for IVDs with CAD, but need manual operations or user-controlled manners to refine the results.

Many other methods have attracted attention because of their potential implications. Chevrefils et al. [12] used a watershed transform and morphological operations to locate regions containing structures of interest. The drawback of this method is the over-segmentation. Neubert et al. [13, 14] proposed an automated approach to extract the 3D segmentations and localization of lumbar and thoracic IVDs using statistical shape analysis and registration of grey level profiles.

Some methods based on machine and deep learning have recently been proposed. Oktay et al. [15] proposed a SVM-based Markov random field method to label the lumbar discs. However, they only detect six discs with their graphical model and require the existence of both T1 and T2 scans to detect the spinal cord. Ghosh et al. [5, 16] achieved the localization of lumbar discs using histogram of oriented gradients along with SVM. Furthermore, a method is proposed to segment all the tissues simultaneously in a lumbar sagittal MRI, using an auto-context approach, instead of any explicit shape features or models. It made strong use of heuristics and information from complementary axial scans. Cheng et al. [17] used a machine-learning based technique to localize and segment the 3D IVDs from MRIs. The IVD localization was done by estimating the image displacements from a set of randomly sampled 3D image patches to the IVD center. The IVDs were segmented by classifying image pixels around disc centers as background or foreground. Kelm et al. [18] combined marginal space learning with a generative anatomical network to detect and label the IVDs. An optional case-adaptive segmentation approach was proposed to segment the IVDs and vertebrae in MRI and CT, respectively.

The presence of intensity inhomogeneities may influence the quality of intensity-based feature extraction. Extraction of information and its service for localization and segmentation of IVDs is important. Compared with other tissues in spinal MRI, IVD has the distinctly characteristic, relatively regular and similar elliptic structure. The extraction of IVD structure information, such as shape and direction, and avoiding interference from peripheral tissues is challenging. The Gabor filter, a windowed Fourier transform, derives from the work of Gabor D. Daugman [19] extended the Gabor filter to two-dimensional (2D) spatial position. Given the biological background and the optimal space and spatial-frequency localization of Gabor filter [20], it is widely used for image process applications [21–23]. When the direction and frequency of the objects in an image are consistent with those of 2D-Gabor filters, the wavelet transformation has a strong response. Since the IVDs in spinal MRI are regular and ellipse-shaped, the recognition of IVDs is possible by transformation of Gabor filtering.

We propose an unsupervised computer-aided IVD localization and segmentation method based on Gabor filter bank, which does not require any training. Among various wavelet bases, the 2D Gabor filters provide good resolution both in temporal and frequency domains, and provide the optimal basis to extract local features because of: (1) Frequency motivation: both multi-resolution and multi-orientation properties of Gabor wavelet are optimal for measuring local spatial frequencies [24]; (2) morphology motivation: it distorts the tolerance space for pattern recognition tasks [24]. The proposed method adopts Gabor filters to extract the structural features of IVDs, and localize and segment the IVDs. The information from the Gabor filter is capable of increasing the accuracy and automation degree for IVD localization and segmentation.

## Methods

### Flow diagram

**Fig. 1:**
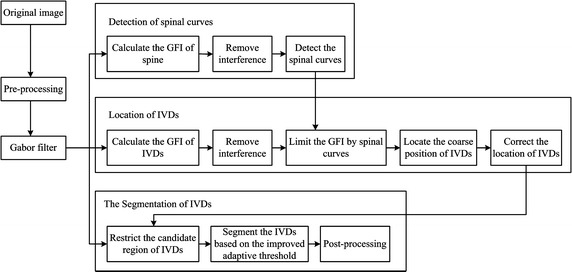
Flow diagram of the proposed method

The flow chart of the proposed method is shown in Fig. 1. It includes the Gabor filter design, detection of spinal curves, localization of IVDs, and segmentation of IVDs. First, a set of 2D-Gabor filters, with different frequencies and directions, are used in image filtering to get a series of Gabor images. Second, Gabor features of the spine are calculated and the spinal curves are detected. Third, the Gabor features images (GFI) of IVDs are calculated and limited by spinal curves. Fourth, the IVD localization is performed by cluster analysis. Correction of localization is done to improve the accuracy of localization results. Last, segmentation of IVDs is performed based on the Gabor filter, localization results, and an improved self-adaptive threshold.

### Setting of the Gabor filter

Effective extraction of the IVD features depends on the setting of the Gabor kernel function. Due to the elliptic shape of IVDs, the parameter $\sigma$ of Gabor kernel is different in horizontal $x$ and vertical $y$ direction. In addition, there is an angle between the IVDs and the horizontal plane. Therefore, the Gabor kernel is defined as [25]:

1 $$\begin{aligned}\psi (x,y,\theta_{\mu } ,\omega_{\nu } ) &= \frac{1}{{2\pi \sigma_{x} \sigma_{y} }}\exp \left\{ { - \frac{1}{2}\left[ {\left(\frac{{x^{\prime}}}{{\sigma_{x} }}\right)^{2} + \left(\frac{{y^{\prime}}}{{\sigma_{y} }}\right)^{2} } \right] + i\omega_{\nu } x^{\prime}} \right\}\\ & \qquad \mu = 0, \ldots ,S - 1, \quad v = 0, \ldots ,K - 1 \end{aligned}$$

where, $x^{\prime} = x\cos \theta_{\mu } + y\sin \theta_{\mu } ,y^{\prime} = - x\sin \theta_{\mu } + y\cos \theta_{\mu }$ represent the spatial locations of pixels. In spatial domain, the parameters $\theta_{\mu }$, $\omega_{\nu }$, and $\sigma$ represent the direction, wavelength, and Gaussian window of Gabor filter bank, respectively.

**Fig. 2:**
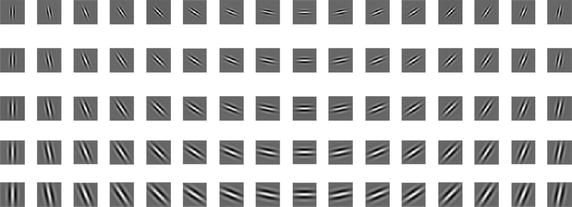
Real components of Gabor filter bank in $S$ directions with $K$ scales ($S = 16,\;K = 5$)

The parameters of Gabor are set as follows: to describe the local features of images, a Gabor filter bank has $S$ directions and $K$ scales (usually $S = 8,\,\,K = 5$) [20]. However, the IVDs have a relatively uniform size and the differences among their angles are smaller. A Gabor filter bank in 16 directions with five scales is used. The upper frequency limit $\omega_{\text {max} } = \frac{\pi }{2}$ is set according to the experience. Since the size of IVDs is relatively uniform, the frequency spacing factor $f = \sqrt[4]{2}$ is set to obtain the effective center frequency. The effective radius of the Gaussian window is expressed as $r_{v} = \frac{2\sqrt 2 \sigma }{{\omega_{v} }}$. According to the characteristics of the IVD (the width occupies at least 25 pixels and the thickness is about half of the width), $\sigma_{x} = \frac{3k}{{\omega_{v} }},\sigma_{y} = \frac{6k}{{\omega_{v} }}$ (where $k = \sqrt {2\ln 2}$). The symmetric Gabor kernel window with the size of $31 \times 31$ is used, based on experiment. Gabor filter bank is shown as Fig. 2.

### Localization of IVDs

#### Detection of spinal curves

**Fig. 3:**
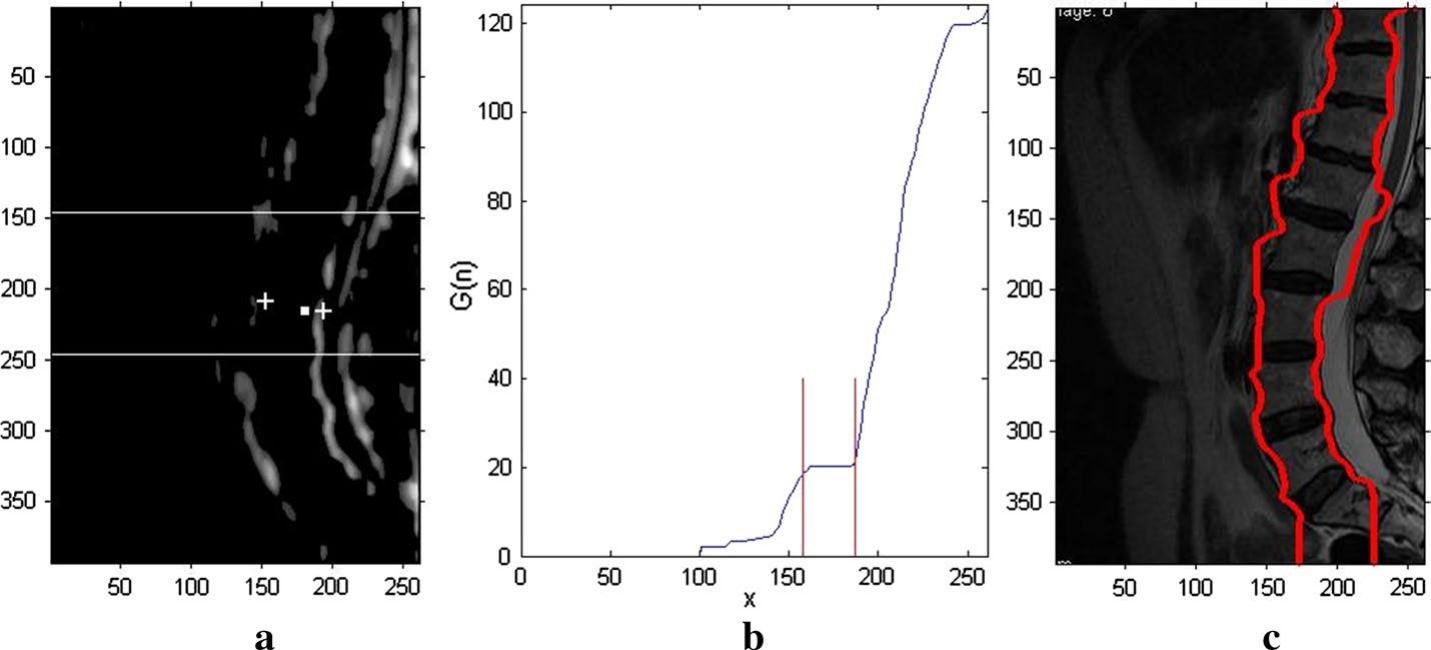
Detection of spinal curves. **a** Spinal GFI $G_{spine}$ The *white point* is the center of the spine, and the *white crosses* are the centers of left and right spinal edges. **b** The sum of the coefficients of the first $n$ columns $G(n)$. **c** The detection of spinal curves

In order to reduce the impact of background on localization and segmentation, the spinal edges are detected to narrow the searching range. Since the spinal curves are nearly vertical, the spinal GFI is obtained by subtracting the GFI of horizontal direction from that of vertical direction. Taking $G_{v,\mu }$ as the Gabor-filtered image with direction $\mu$ and scale $v$, the spinal GFI $G_{spine}$ can be described as Eq. (2):

2 $$G_{spine} = \overline{{\sum\limits_{{\nu \in C,\mu \in U_{1} }} {G_{\nu ,\mu } } }} - \overline{{\sum\limits_{{\nu \in C,\mu \in U_{2} }} {G_{\nu ,\mu } } }}$$

where $v \in C = \left\{ {0,1, \ldots ,S - 1} \right\}$, $\mu \in U = \left\{ {0,1, \ldots ,K - 1} \right\}$, $U_{1} = \left\{ {0,1,2,3,4,5,14,15} \right\}$, and $U_{2} = \left\{ {7,8,9,10,11} \right\}$ represent the directions close to the vertical and horizontal planes, respectively. Obviously, the negative values in $G_{spine}$ are not the edge information of spine. Then, the $G_{spine}$ is processed as Eq. (3).

3 $$G_{spine} (x,y) = \left\{ \begin{array}{ll} G_{spine} ( x,y),&\quad G_{spine} ( x,y ) \ge 0 \\ 0,&\quad G_{spine} ( x,y ) < 0 \\ \end{array} \right.$$

4 $${G\left( n \right) = \sum\limits_{x = 1}^{n} {\sum\limits_{y = (M - p)/2}^{(M + p)/2} {G_{spine} (x,y)} } ,} \quad {n = 1 \ldots } N$$

where $G(n)$ is the sum of the GFI for the first $n$ column (Fig. 3b), and $N$ is the column number of the image. Since the middle of the spine is nearly straight, the $p$ rows in the middle of the MRI images are selected to detect the spinal edges. As shown in Fig. 3b, the curve of $G(n)$ is approximately in the horizontal plane in the middle of the spinal region (the middle of the two white lines in Fig. 3a). The process include: first, the centers of the left and right spinal edges (the white crosses in Fig. 3a) are obtained by searching the crossing points for $G_{spine}$ and center of spine (the white point in Fig. 3a from center to both sides). Second, the search range is limited between the two white lines in Fig. 3a. Third, the left and right spinal edges are obtained by searching the corresponding region from above the centers of left or right spinal edges to both sides, respectively. The Fig. 3c shows the result of spinal edges.

#### Localization of IVDs

**Fig. 4:**
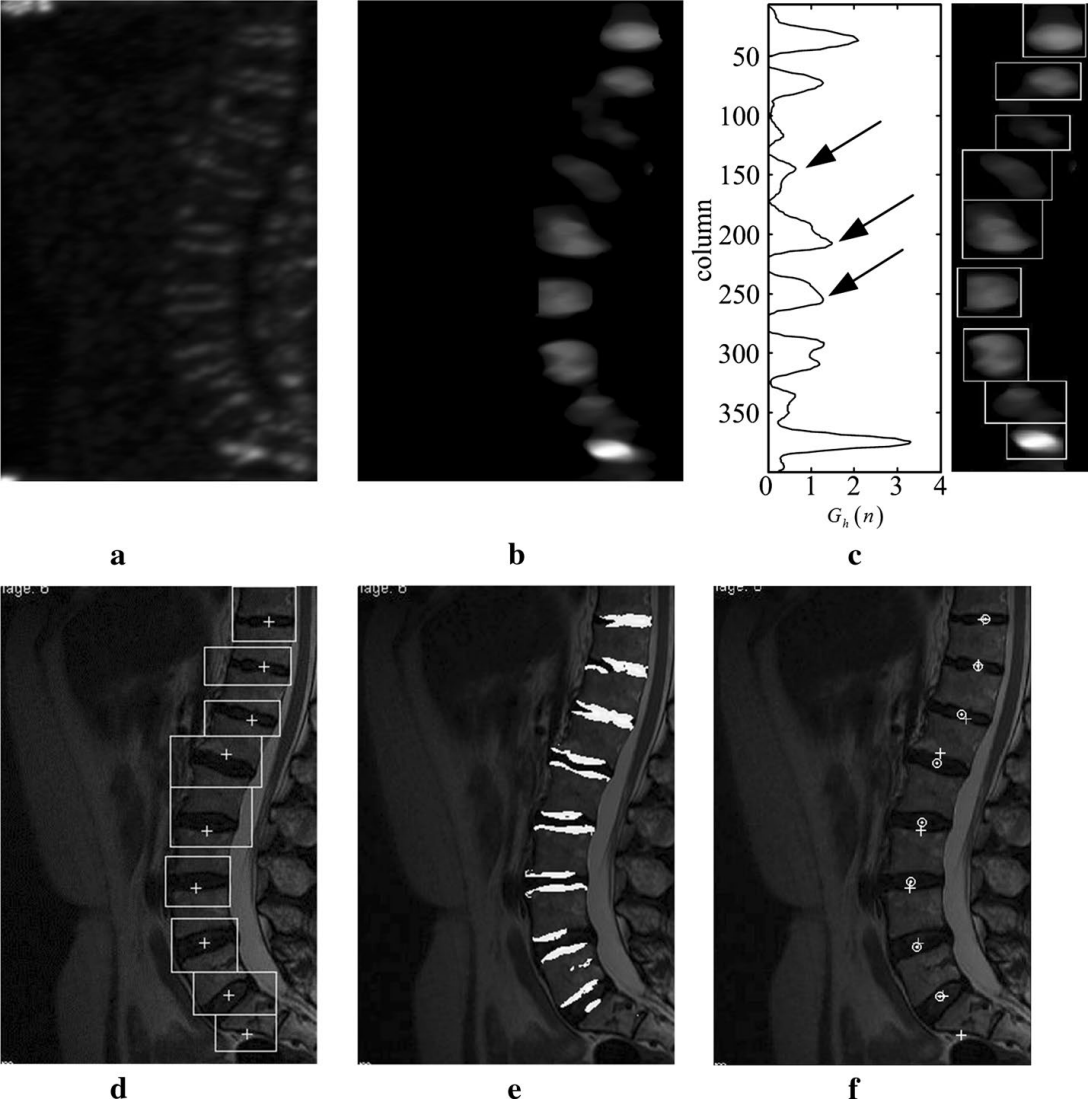
IVDs localization information based on the Gabor filter. **a** IVDs GFI. **b** IVDs information images $G_{Mdisc}$. **c** Horizontal cumulative curve $G_{n} (n)$. **d** IVD areas delineation based on localization results. **e** IVD edges determined by Gabor feature shown as the superposition of the original image and the binary image. **f** Comparison of localization result before (*cross*) and after (*circle*) correction

The localization of IVDs is based on GFI information. GFI for localization of IVDs is calculated similar to the process for spines, but the directions of $U_{1}$ and $U_{2}$ are different. According to the anatomical knowledge, the angle between the long axis of the IVD and the horizontal line of MRI is almost ± 30°. Therefore, $U_{1} = \left\{ {7,8,9,10,11} \right\}$ and $U_{2} = \left\{ {1,2,3,4,5} \right\}$ represent directions close to the long axis of IVDs and close to the vertical directions, respectively. The IVDs information image $G_{Mdisc}$ (Fig. 4b) is obtained by median filtering (elliptical filter template, long axis: 44 pixels, minor axis: 17 pixels) on the IVDs GFI (Fig. 4a). The searching range of IVDs is narrowed by spinal curves.

The process of localization needs some a priori information. The centroid of the IVD is the locating point. In order to obtain the coordinate value of the locating point $(X_{IVD} ,Y_{IVD} )$, two aspects of a priori anatomical knowledge are adopted: I)Abscissa $x$ of most IVDs is roughly located in the same vertical line, except for the last few ones that lean slightly to the right; II) The offsets of vertical $y$ range from 20 mm to 50 mm (25 pixels to 60 pixels in this study). The information of local maximum of $G_{Mdisc}$, combined with a priori knowledge, is used for coarse localization. In this process, correction of centre-of-gravity shift is also performed to improve the localization accuracy in case of severe spinal curvature. The process includes:

Step 1: Calculate the horizontal cumulative curve $G_{h} \left( n \right)$ and the candidate vertical coordinate values. $G_{h} \left( n \right)$ is obtained by adding each row with Eq. (5), Where $M$ is the number of rows of the image. The coordinates of local maximum values are the candidates.
  5 $${G_{h} \left( n \right) = \sum\limits_{x = 1}^{N} {G_{Mdisc} \left( {x,n} \right),} } \quad {n = 1 \ldots M}$$
Step 2: Calculate the coarse $Y_{IVD}$. According to the a priori information II, the closer points merge into one. The $Y_{IVD}$ is achieved after removing the points which do not meet the a priori information II.
Step 3: Calculate the coarse $X_{IVD}$. The horizontal coordinate $X_{IVD}$ is calculated by a process similar to that for $Y_{IVD}$, but the regions of calculation are different. The mid-values of adjacent two $Y_{IVD}$ are taken as boundaries to intercept the IVD part. The same action is performed as Eq. (6) in columns between the two boundaries. The $X_{IVD}$ is obtained based on the a priori information I.
  6 $${G_{v} \left( n \right) = \sum\limits_{y = 1}^{M} {G_{Mdisc} \left( {n,y} \right),} } \quad {n = 1 \ldots N}$$
Step 4: Calculate the boxes of IVDs to correct the coarse($X_{IVD}$, $Y_{IVD}$). Based on the results of Step 3, the rectangular regions of IVDs (Fig. 4d) are found by the boundary of the local peak of $G_{h} \left( n \right)$ (Fig. 4c) and $G_{v} \left( n \right)$. The upwards and downwards edges of the box in Fig. 4c are calculated by the minimum points and zero points of $G_{h} \left( n \right)$. Then the left and right edges of the box are calculated by the minimum points and zero points of $G_{v} \left( n \right)$ limited by upwards and downwards edges. Figure 4c shows the boxes in $G_{Mdisc}$. Figure 4d shows the boxes in the original image. The angles of IVDs are obtained by analyzing the GFIs information in the rectangular regions.
Step 5: Calculate the accurate center of IVDs $(X_{IVD} ,Y_{IVD} )$. The binary image of $G_{Mdisc}$ is calculated in the boxes obtained in step 3. Figure 4e shows the superposition of the original image and the binary image. Subsequently, the corrected localization of IVDs is obtained by calculating the centroid of the binary image. As shown in Fig. 4f, the white circles are the final localization results $(X_{IVD} ,Y_{IVD} )$.

### Segmentation of IVDs

**Fig. 5:**
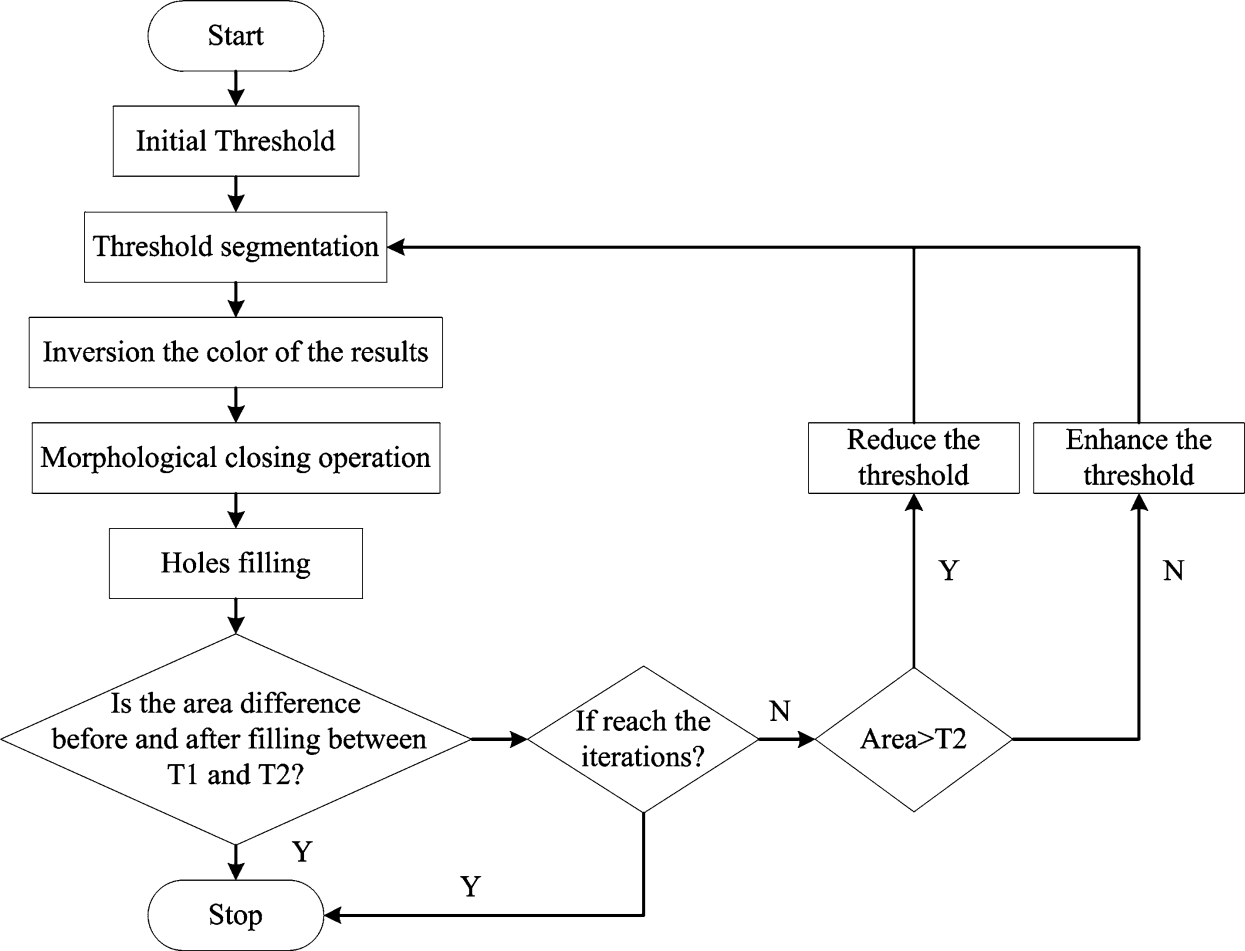
Flowchart for the adaptive local threshold based on the iteration
Step 3: Calculate the coarse segmentation of the IVD. The coarse segmentation results of IVDs are used in steps 2 to 5, in Fig. 5, with an adaptive local threshold $T_{IVD}$.
Step 4: Post-process for IVD segmentation. The coarse segmentation result may contain holes, non-smooth areas, and superfluous portions. In order to obtain the accurate segmentation, a morphological operator is used: holes filling, erosion, dilation, and five foreground pixels on the background pixel indicate that the pixel is the foreground pixel. When a segmentation result has many connected regions, the largest region is retained.

Combining the localization results and spinal curves,the segmentation of IVDs is performed based on GFIs with an adaptive threshold. The main steps include:

Step 1: Delineate the candidate region of the IVD. The initial candidate region of each IVD for localization is comprised of the spine curves and the rectangular regions of IVDs (Fig. 4d). In this region, the average of GFIs of different frequencies $\bar{G}_{\mu }$ is calculated in the direction $\mu$. The maximum $\bar{G}_{\mu }$ can reflect the boundary of the IVD. The $\bar{G}_{\mu }$ in the direction $\mu$ with $S$ different frequencies is defined as Eq. (7).
  7 $$\bar{G}_{\mu } \left( {x,y} \right) = \frac{1}{S}\sum\limits_{v \in C} {G_{\nu ,\mu } }$$
  The binary image of maximum $\bar{G}_{\mu }$ is obtained using the Otsu method. The up and down boundaries of the candidate region are comprised of the spine curves. The left and right boundaries are comprised of the fitting curve of the outer boundaries of the binary image of maximum $\bar{G}_{\mu }$.
Step 2: Calculate the adaptive local threshold $T_{IVD}$. Due to the ambiguous boundary and diverse shapes of IVDs, global threshold is not appropriate to segment the IVDs. Therefore, the local threshold ($T_{IVD}$) of each IVD is adopted to segment the IVDs. As shown in Fig. 5, $T_{IVD}$ is obtained by self-adaption iteration and used for segmentation of IVDs. The initial threshold is calculated by the Otsu method. $A_{1}$ is the difference between the area of candidate region of the IVD and the area of the binary image of maximum $\bar{G}_{\mu }$. $A_{2}$ is 1/2 area of the binary image of maximum $\bar{G}_{\mu }$. $T_{1}$ and $T_{2}$ are $\hbox{min} (A_{1} ,A_{2} )$ and $\hbox{max} (A_{1} ,A_{2} )$ area, respectively. The number of iterations is 30.

## Results and discussion

### Ethic statement

This study is approved by the Ethics Review Board of the Third Military Medical University, Chongqing, China. All records, information, and images were anonymized and de-identified prior to analysis. All patients or their legal representatives signed the written informed consent.

### Image acquisition

This dataset was composed of mid-sagittal T2-weighted (T2WI) images from 37 patients with LBP for more than 6 months. In total, 278 IVDs (T11/T12 ~ L5/S1) of MRI images were utilized for validating the proposed localization methods. All MRI images were supplied by Xinqiao Hospital, The Third Military Medical University, China. MRI was performed with a 1.5-T Signa system (General Electric Company, Milwaukee, America), and the parameters of the T2WI were: TR: 3000 ms; TE: 100 ms; FOV: 30 × 30; and number of sagittal sections: 9.

### Results of localization

In this study, 278 IVDs of MRI images from 37 patients were utilized to validate the proposed method. All methods were implemented in MATLAB R2010b. Of a total of 283 IVDs, 278 IVDs were located by our method, with only five IVDs seen outside of the localization.

**Fig. 6:**
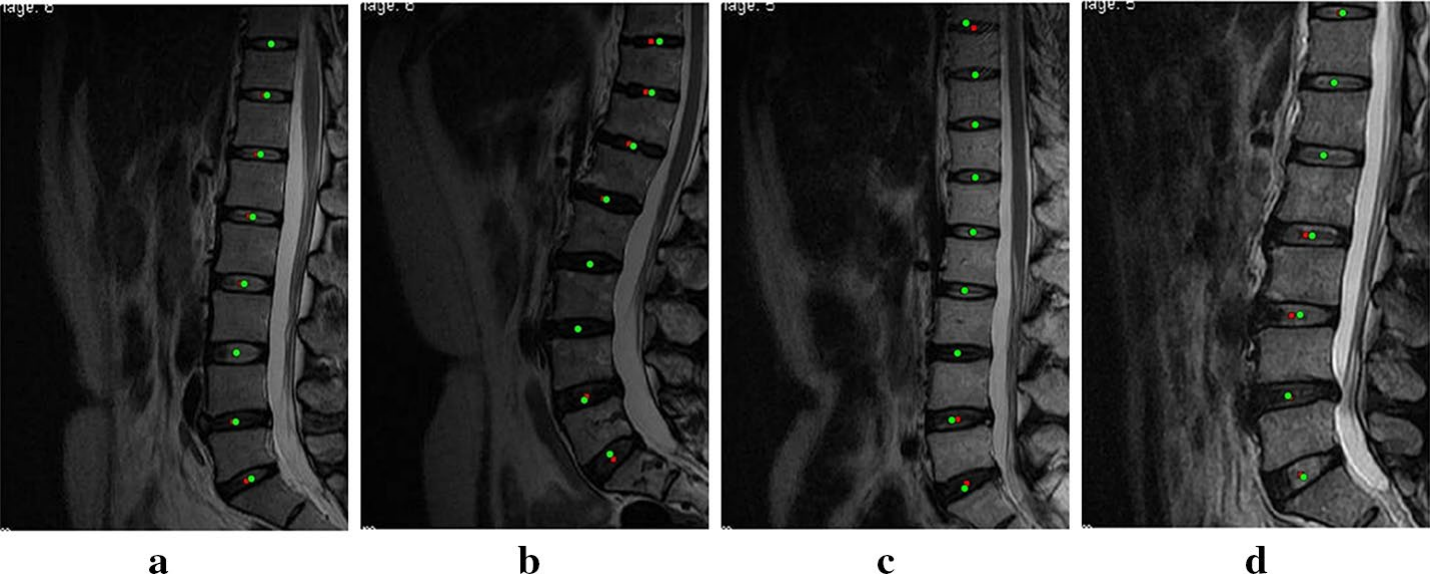
Comparison of the localization results of our method and the manual method. The *red squares* are the results of the manual method, and the *green circles* are the results of our method. **a**–**d** contain the typical cases including normal patient, herniation and IVD degeneration, different intensity and interference, and different spine curvature

We compared the localization with our method and the manual method. As Fig. 6 shows, the red squares are the results of our method and the green circles are those of the manual method. The four MRIs shown in the Fig. 6a–d contain the typical cases including normal patient, herniation and IVD degeneration, different intensity and interference, and different spine curvature. These figures show that the results of our method have good agreement with those of the manual operation.

**Fig. 7:**
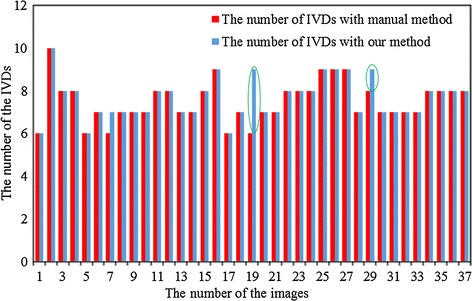
Comparison of the numbers of localized IVDs between our method and the manual method. The *green ellipses* are the IVDs extra localization

To evaluate the performance of the method quantitatively, localization accuracy $Acc$ was used. $Acc$ is the percentage of automatic IVD centers which visually lie inside the IVD. It is defined as:

8 $$Acc = \frac{{Disc_{AutoTure} }}{{Disc_{Auto} }} \times 100~\%$$

where $Disc_{AutoTure}$ is the number of correct results of automatic localization of IVDs, $Disc_{AutoTure}$ is the number of all results of automatic localization of IVDs. The results show that a high localization accuracy $Acc$ of 98.23 % could be achieved with the method proposed. The comparison between the number of IVDs with our localization method and those with manual method is shown in Fig. 7.

**Fig. 8:**
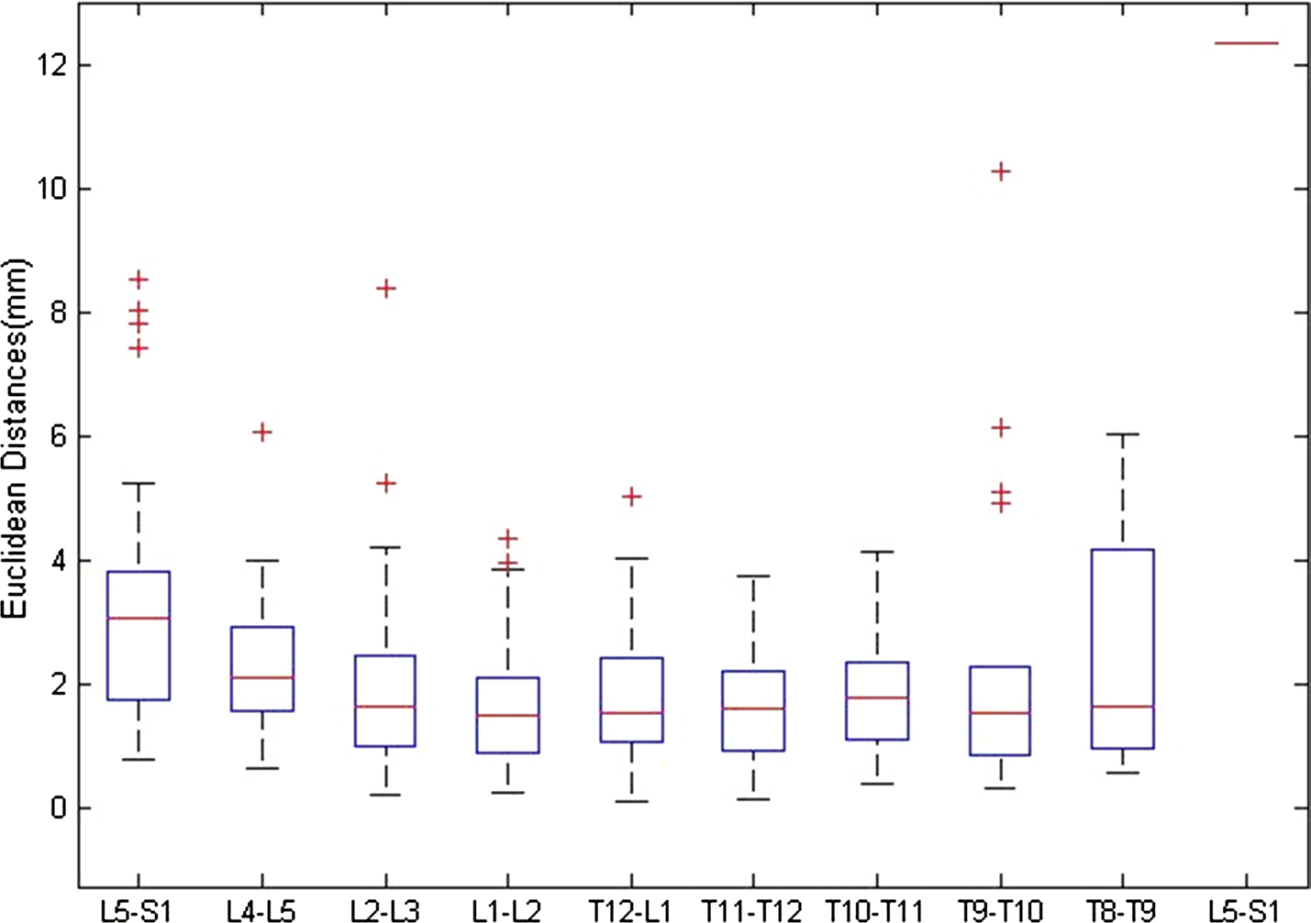
*Boxplot* of the Euclidean distances between the structure center localized with our method and the corresponding manual method for 37 MR images in the dataset

The Euclidean distances between the structure center located by our method and the corresponding manual method were also calculated. Three surgical specialists delineated the contours of each structure manually. Based on the contours, the computer calculated the centers of the IVDs with a snake-based method [26]. The boxplot of the Euclidean distances for the spine structures in 37 images is shown in Fig. 8. The median, top, and bottom lines of the box represent the 50th, 25th, and 75th percentiles, respectively, and the pluses are the statistical outliers. Although there are 14 off-group points from 278 in all cases, these points are all in IVDs and not far from the results of the manual operation. Table 1 shows the average localization error from reference annotation to the localized positions.

**Table 1:** Average localization error (in mm) from reference annotation to the localized positions

Disc label	Mean ± STD	Median	Max	Min
L5-S1	3.2258 ± 2.0326	3.0415	8.5399	0.7835
L4-L5	2.3505 ± 1.0514	2.0809	6.0460	0.6439
L3-L4	2.0117 ± 1.6307	1.6358	8.3857	0.2093
L2-L3	1.6300 ± 0.9993	1.4974	4.3328	0.2453
L1-L2	1.7608 ± 1.0563	1.5384	5.0205	0.0899
T12-L1	1.5694 ± 0.9160	1.5824	3.7216	0.1191
T11-T12	1.8817 ± 1.0163	1.7635	4.1179	0.3896
T10-T11	2.4007 ± 2.6023	1.5336	10.2740	0.3162
T9-T10	2.5622 ± 2.2372	1.3515	6.0186	0.5505
T8-T9	12.3583	–	–	–
All	2.1327 ± 1.6337	2.0786	12.3583	0.0899

**Fig. 9:**
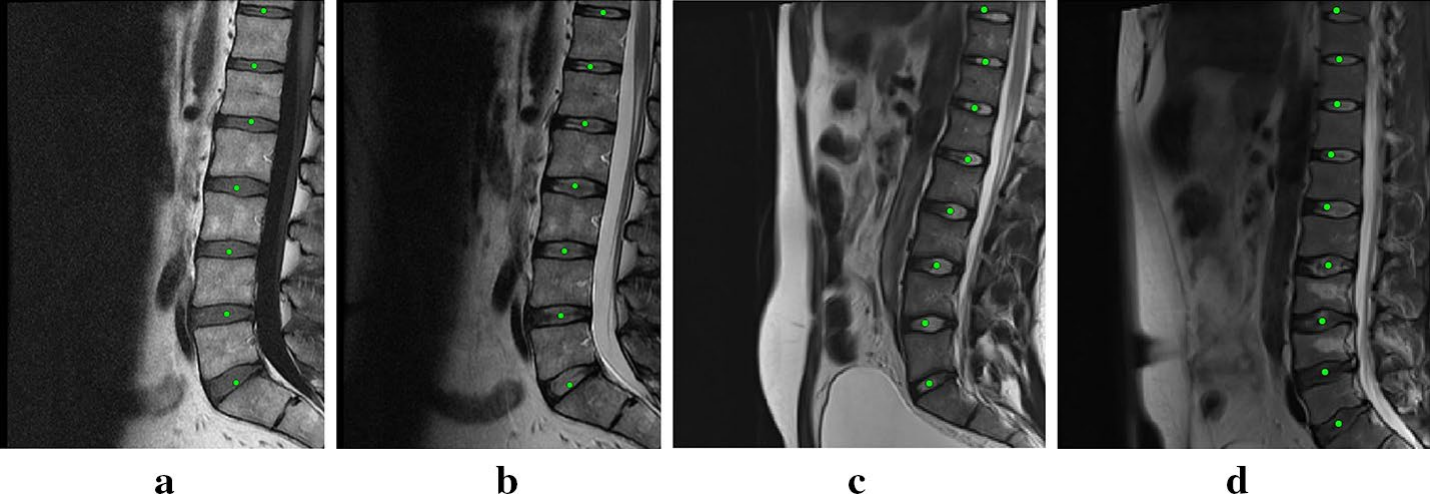
The localization results of the SpineWeb database. **a**, **b** and **c**, **d** are from different databases and subjects

In order to test the validity of our method in the other dataset, experiments were performed on the publicly available SpineWeb database which is available for public access at http://spineweb.digitalimaginggroup.ca/spineweb. The localization results of SpineWeb database are shown in Fig. 9. Figure 9a–d are from different databases and subjects. The mean and standard deviation of localization using Dataset seven of SpineWeb was 2.60 ± 1.90 mm.

### Results of segmentation

**Fig. 10:**
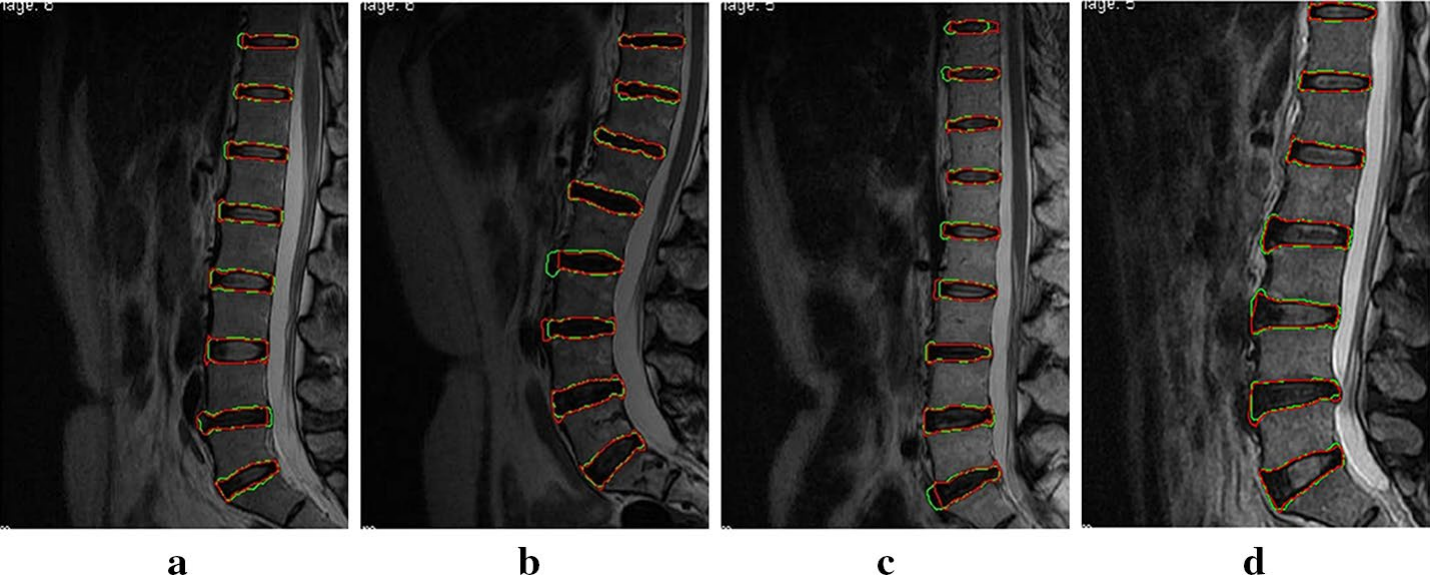
The comparison between the segmentation results of our method and those of the manual method. The *red contour* shows the results of the manual method, and the *green contour* shows the results of our method. **a** is the normal case. **b** is the case with more serious curvature and IVD degeneration. **c**–**d** are the case with higher intensity, herniation and IVD degeneration

The same cases were used to validate the segmentation. Figure 10 shows the segmentation results with our proposed method and manual operation. The green contours are the results of our method, and the red contours are the results of the manual method. The figure shows that the results of the two methods are highly consistent for normal cases. Figure 10c–d show the results for cases with higher intensity, herniation, and IVD degeneration. As shown in Fig. 10b, in cases with more serious curvature and IVD degeneration, L2-L3 are over-segmented and L3-L4 are slightly under-segmented. Although the spinal curves were deviated, overall segmentation results of IVDs were satisfactory.

To evaluate the performance of the segmentation method quantitatively, Sensitivity ($Sen$), Specificity ($Spe$), and Dice Similarity Index *(DSI)* were calculated [12–15]. The $Sen$, $Spe$, and *DSI* were defined as:

9 $$Sen = \frac{M \cap A}{M}$$

10 $$Spe = \frac{I - M \cup A}{I - M}$$

11 $$DSI = \frac{{2\left| {M \cap A} \right|}}{\left| M \right| + \left| A \right|}$$

where $M$ is the area of the manually segmented IVD, $A$ is the area of the segmented IVD experimentally, and $I$ is the MRI image.

The index analysis of 37 MRIs is shown in Table 2. The overall average values of the *DSI* with and without GFIs is 0.9237 and 0.8899, respectively. The index values show that the results with GFIs restriction have significant improvements compared with those without GFIs restriction. The results of the proposed method have good agreement with those of the manual segmentation. There was reduced probability of over- or under-segmentation.

**Table 2:** The comparison of DSI, Sen, and Spe with and without GFIs

Methods	Labels of IVDs	Mean DSI	Mean Sen	Mean Spe
With GFIs	L5-S1	0.9349	0.9513	0.9986
	L4-L5	0.9320	0.9304	0.9983
	L3-L4	0.9469	0.9541	0.9985
	L2-L3	0.9352	0.9652	0.9984
	L1-L2	0.9389	0.9608	0.9987
	T12-L1	0.9376	0.9558	0.9989
	T11-T12	0.9332	0.9451	0.9990
	T10-T11	0.8551	0.9150	0.9993
	T9-T10	0.8997	0.9107	0.9996
Without GFIs	L5-S1	0.8067	0.7708	0.9991
	L4-L5	0.8911	0.8667	0.9985
	L3-L4	0.9119	0.9269	0.9981
	L2-L3	0.9141	0.9658	0.9979
	L1-L2	0.8996	0.9475	0.9982
	T12-L1	0.9197	0.9774	0.9985
	T11-T12	0.9233	0.9662	0.9988
	T10-T11	0.8450	0.8755	0.9987
	T9-T10	0.8975	0.9908	0.9985

## Discussion and conclusion

The proposed method achieves high accuracy without the need of user interaction, technologist point, or coronal or axial slices. This benefit comes from the shape-recognition ability of the method based on Gabor filter bank. GFIs reduce the localization error by extracting the target information correctly, and correction operation reduces the impact of inaccurate IVD localization by taking all candidate points into account. In the segmentation part, the adaptive threshold based on the GFI is proposed. The GFI limits the candidate regions, which improves the accuracy of the segmentation results.

The localization and segmentation of the proposed method takes almost 10 s (the manual segmentation with a snake-based method [26] takes almost 5 min). Eight seconds are required for one image to complete the localization process, and only 2 s are required to complete the segmentation part. The main contribution to the computation time is by wavelet transformation (about 7.5 s). With the development of computer technology, our method is likely to satisfy the requirements of real-time clinical application.

The Gabor filter bank makes use of the direction and morphology of the IVDs. Due to the interference in direction and morphology in some MRIs, few IVDs were localized outside of the localization results. The results were not significantly affected by the extra localization because recognition techniques, such as areas and morphology, were added to the segmentation method. However, the localization accuracy is likely to increase even further with the use of the texture feature in this method. This method was verified with 278 IVDs from 37 patients. Further verification and improvement of the method is warranted in future studies. Additional studies with large sample sizes are necessary to use GFIs for exploring the characters of IVDs and segmentation. We plan to carry out more studies with large sample sizes. Further work also includes classifying the degree of IVD degeneration using the segmentation and curvature results. Additionally, the GFI information will be used to detect curvature of spines more accurately so diseases of the spine can be accurately diagnosed.

In conclusion, we proposed a localization and segmentation method for IVDs based on Gabor wavelet. In contrast to traditional methods, Gabor filtering for shape-representation is proposed. The results and quantitative evaluation show that this method has a high accuracy compared with manual operation. It does not need supervision or training with large datasets before use. Therefore, this method will be useful in computer-aided diagnosis of the disease and computer-assisted spine surgery.

## References

[1] Ract I, Meadeb JM, Mercy G, Cueff F, Husson JL, Guillin R (2015). A review of the value of MRI signs in low back pain. Diagn Interv Imaging.

[2] Dagenais S, Galloway EK, Roffey DM (2014). A systematic review of diagnostic imaging use for low back pain in the United States. Spine J.

[3] Lzzo R, Popolizio T, D’Aprile P, Muto M (2015). Spinal pain. Eur J Radiol.

[4] Siemund R, Thurnher M, Sundgren PC (2015). How to image patients with spine pain. Eur J Radiol.

[5] Ghosh S, Malgireddy MR, Chaudhary V, Dhillon G. A new approach to automatic disc localization in clinical lumbar MRI: combining machine learning with heuristics. 9th IEEE International Symposium on Biomedical Imaging (ISBI). 2012;114–117.

[6] Alomari RS, Corso JJ, Chaudhary V (2011). Labeling of lumbar discs using both pixel-and object-level features with a two-level probabilistic model. IEEE Trans Med Imaging.

[7] Michopoulou SK, Costaridou L, Panagiotopoulos E, Speller R, Panayiotakis G, Todd-Pokropek A (2009). Atlas-based segmentation of degenerated lumbar intervertebral discs from MR images of the spine. IEEE Trans Biomed Eng.

[8] Peng ZG, Zhong J, Wee W, Lee JH. Automated vertebra detection and segmentation from the whole spine MR images. 2005 27th Annual International Conference of the IEEE Engineering in Medicine and Biology Society. 2005;2527–2530. 10.1109/IEMBS.2005.1616983 17282752

[9] Castro-Mateos I, Pozo JM, Lazary A, Frangi AF (2014). 2D segmentation of intervertebral discs and its degree of degeneration from T2-weighted magnetic resonance images. Medical imaging 2014. Comput Aided Diagn.

[10] Haq R, Aras R, Besachio DA, Borgie RC, Audette MA (2015). 3D lumbar spine intervertebral disc segmentation and compression simulation from MRI using shape-aware models. Int J Comput Assist Radiol Surg.

[11] Law MWK, Tay K, Leung A, Garvin GJ, Li S (2013). Intervertebral disc segmentation in MR images using anisotropic oriented flux. Med Image Anal.

[12] Chevrefils C, Cheriet F, Grimard G, Aubin C (2007). Watershed segmentation of intervertebral disk and spinal canal from MRI images. Image Anal Recognit.

[13] Neubert A, Fripp J, Engstrom C, Schwarz R, Lauer L, Salvado O (2012). Automated detection, 3D segmentation and analysis of high resolution spine MR images using statistical shape models. Phys Med Biol.

[14] Neubert A, Fripp J, Engstrom C, Walker D, Weber MA, Schwarz R (2013). Three-dimensional morphological and signal intensity features for detection of intervertebral disc degeneration from magnetic resonance images. J Am Med Inform Assoc.

[15] Oktay AB, Akgul YS (2013). Simultaneous localization of lumbar vertebrae and intervertebral discs with SVM-based MRF. IEEE Trans Biomed Eng.

[16] Ghosh S, Chaudhary V (2014). Supervised methods for detection and segmentation of tissues in clinical lumbar MRI. Comput Med Imaging Graph.

[17] Chen C, Belavy D, Yu W, Chu C, Armbrecht G, Bansmann M, Felsenberg D, Zheng G (2015). Localization and segmentation of 3D intervertebral discs in MR Images by data driven estimation. IEEE Trans Med Imaging.

[18] Kelm BM, Wels M, Zhou SK, Seifert S, Suehling M, Zheng Y, Comaniciu D (2012). Spine detection in CT and MR using iterated marginal space learning. Med Image Anal.

[19] Daugman JG (1985). Uncertainty relation for resolution in space, spatial frequency, and orientation optimized by two-dimensional visual cortical filters. J Opt Soc Am.

[20] Daugman JG (1988). Complete discrete 2-D Gabor transforms by neural networks for image analysis and compression. Transact Acoust Speech Signal Process.

[21] Radman A, Jumari K, Zainal N (2014). Iris segmentation in visible wavelength images using circular gabor filters and optimization. Arab J Foren Eng.

[22] Hacihaliloglu I, Rasoulian A, Rohling RN (2014). Local phase tensor features for 3-D ultrasound to statistical shape + pose spine model registration. IEEE Trans Med Imaging.

[23] Shu T, Zhang B (2015). Non-invasive health status detection system using gabor filters based on facial block texture features. J Med Syst.

[24] Ji P, Jin L, Li X. Vision-based vehicle type classification using partial Gabor filter bank. 2007 IEEE International Conference on Automation and Logistics. 2007;1037–1040.

[25] Lee TS (1996). Image representation using 2D Gabor wavelets. IEEE Trans Pattern Anal Mach Intell.

[26] Zhu X, Zhang P, Shao J (2011). A snake-based method for segmentation of intravascular ultrasound images and its in vivo validation. Ultrasonics.

